# Redesigning traditional grains: a functional approach to protein quality using Kodo millet and chickpeas

**DOI:** 10.3389/fnut.2025.1638750

**Published:** 2025-10-28

**Authors:** Deeksha Rana, Renuka Aggarwal, Harpreet Kaur, Aditi Sewak, Kiran Bains, Inderpreet Kaur Dhaliwal

**Affiliations:** ^1^Department of Food and Nutrition, Punjab Agricultural University, Ludhiana, Punjab, India; ^2^Department of Plant Breeding and Genetics, Punjab Agricultural University, Ludhiana, Punjab, India

**Keywords:** chickpea, Kodo millet, nutrition composition, recommended dietary allowance, value-added products, sensory evaluation

## Abstract

**Introduction:**

Diversifying staple diets with nutritionally dense crops like millets offers a viable strategy to combat hidden hunger and undernutrition. Studies suggest that including coarse cereals like millets can compensate for amino acid deficiencies. Kodo millet is rich in essential amino acids such as lysine, threonine, valine, and sulfur-containing amino acids, with a leucine-to-isoleucine ratio of approximately 2.0. Therefore, the present investigation was carried out to formulate and nutritionally evaluate millet-pulse-based food products by replacing traditional cereals.

**Methods:**

Food products, such as “crispy crunch” and “nutrimilletvita,” were formulated using Kodo millet (IPS 178), wheat (PBW 826), rice (PR 131), and chickpea in different proportions. For “crispy crunch,” the ingredients were supplemented in the ratio of 50:30:20 percent, while “nutrimilletvita” was prepared with 50 percent supplementation of each of Kodo millet and chickpea. The developed food products were tested for their acceptability and nutritional composition against the traditional cereal.

**Results:**

The overall acceptability of formulated crispy crunch and nutrimilletvita was 7.54 and 7.80, respectively, which was comparable with the wheat- and rice-based product and wheat-based product, respectively. These products had a substantial amount of protein (10.01–18.31 g/100 g) and lysine (3.96–4.06 g/100 g) with considerable *in vitro* protein digestibility (73.7–78.3%) and protein digestibility amino acid score (0.39–0.45). The products, when stored for 3 months, were found to be organoleptically acceptable for 45 days. The developed products were cost-effective and nutritionally superior when compared with their traditional and market counterparts.

**Conclusion:**

The study concluded that consumption of one serving of developed kudo millet chickpea-based products met 22–41% of the recommended dietary allowance (RDA) for protein and 46.1–79.5% of the lysine requirements of school-going children (7–12 years). Hence, the developed Kodo millet–chickpea combination can be effectively used as a replacement for wheat/rice for preparing various food products with good-quality protein.

## 1 Introduction

Every manifestation of childhood malnutrition, such as undernutrition (wasting, stunting, and underweight), micronutrient deficiencies, coupled with overweight and obesity, represents the triple burden of disease, particularly affecting low- and middle-income countries, and is a major contributor to poor health globally. It poses a significantly negative impact, being a major impediment to individual development, and hinders the achievement of full human potential (1). In 2022, global estimates indicated that 149 million children under the age of 5 years were stunted, nearly 45 million experienced wasting, around 340 million faced micronutrient deficiencies, and approximately 37 million were overweight or obese (2, 3). However, approximately 45% of deaths among children under 5 years worldwide can be attributed to undernutrition (4).

Child and maternal undernutrition remain a significantly persistent public health concern worldwide, especially in India, bearing the responsibility of nearly 15% of the country’s disease burden throughout (5). In addition to improvements in the availability of food, great segments of the population, especially vulnerable groups of the society (infants, young children, and women of reproductive age), remain susceptible to protein-energy malnutrition. However, inadequate intake of quality protein, critically in terms of amino acid composition, remains a continuing contributor to this predicament. Approximately 80% of children under 5 years in India are unable to meet minimum daily protein requirements when adjusted for amino acid composition. This shortfall contributes significantly to prevalent subclinical deficiencies and associated functional impairments (6). Due to plant-based dominated food preferences of major strata of the Indian population, the biological value of dietary protein remains suboptimal, characterized by an inherent deficiency in essential amino acids, notably lysine (7).

Lysine is an essential amino acid, crucial for collagen synthesis, absorption of calcium in human metabolism, production of various hormones, maintaining functionality of the immune system, and retention of nitrogen in the body, particularly during periods of rapid growth (infancy and adolescence) (8). It is often the first limiting amino acid in cereal-based diets, prepared primarily with wheat and rice as staple foods for most Indians. Unlike animal proteins, offering a balanced amino acid profile, plant-based proteins are commonly deprived of a good amino acid balance. Being characteristically low in lysine, cereal proteins may be complemented with legumes such as chickpeas, which are deficient in sulfur-containing amino acids like methionine and cysteine. This combination can complete the amino acid profile, resulting in the consumption of good-quality protein (9). However, an imbalance among these amino acids leads to incomplete protein synthesis, which further impairs lean body mass deposition and may compromise immune defense and cognitive function in children.

Children bearing chronic lysine deficiency may not suffer from evident protein-calorie malnutrition, but they may experience the condition referred to as “hidden hunger,” which is a form of micronutrient and amino acid insufficiency posing serious long-term consequences. Literature indicates that inadequate consumption of lysine among school-going children is associated with obstruction in linear growth, stunting, and increased susceptibility to infections owing to reduced hemoglobin levels. Sometimes, this deprivation may have consequences, such as delayed sexual maturation and impaired neurodevelopmental outcomes (10, 11). Furthermore, lysine deficiency can hinder serotonin metabolism, thereby becoming causative of behavioral and cognitive turbulences (12). In this regard, dietary intake of quality protein by children becomes a critical determinant of their nutritional status than the quantity of protein alone.

Nonetheless, despite the implementation of several nutrition support programs in the country, such as the Mid-Day Meal Scheme and Integrated Child Development Services (ICDS), their effectiveness is inhibited due to the intake of poor-quality dietary protein in the provided meals. The National Family Health Survey-5 (13) reports suggested a minimal improvement in stunting and wasting indicators among Indian children over the past decade, emphasizing the insufficiency of current dietary interventions in addressing indispensable nutrient shortfalls. Achieving Sustainable Development Goal 2 (SDG-2), which calls for ending all forms of malnutrition by 2030, is not just based upon caloric sufficiency but also a transformation in dietary protein sources and quality.

In this regard, there has been an increasing scientific interest toward studying millets, especially underutilized minor millets, for their potential role in food and nutritional security. Millets are proficient in nutrients like dietary fiber, polyphenols, and micronutrients while they are resilient to climate stress. Among these, Kodo millet (*Paspalum scrobiculatum*) is distinguished due to its superior nutritional profile and traditional acceptance in numerous Indian communities, containing approximately 8–9% protein, a significantly higher fiber content (~9%) with a promising amino acid composition than major cereals (rice or wheat) (14, 15). Prominently, Kodo millet contains higher lysine levels than most cereals, although not sufficient on its own to completely meet lysine requirements of the body (16). Therefore, it becomes highly complementary when combined with lysine-rich legumes, such as chickpeas.

Chickpeas (*Cicer arietinum*) are a nutritionally well-acknowledged legume with a protein content ranging from 18 to 22% with a high lysine concentration. However, being deficient in methionine and cysteine, their independent effectiveness as a complete protein source is restrained (17). The combination of Kodo millet and chickpeas has the potential to overcome the mutual limitations due to their complementary amino acid profiles, resulting in a superiorly balanced and complete protein intake. This strategy can be ideal for addressing the protein quality gaps in vegetarian diets. Additionally, such combinations can provide a rich matrix of micronutrients as exemplified by iron, calcium, folate, and zinc, which are often concurrently deficient in regular diets of children, particularly in low-resource settings.

The functional and nutritional symbiosis of Kodo millet and chickpeas proposes a novel scope for the development of cost-effective, culturally suitable, protein-rich food products for children. Nonetheless, research evidence has assessed the nutritional benefits of millets or legumes independently, while few have explored their integrated potential in addressing lysine deficiency and improving the intake of quality protein among Indian children. Therefore, the current research aims to bridge that gap by scientifically formulating and evaluating value-added functional food products based on Kodo millet and chickpeas, specifically designed for school-going children.

Further, the thermo-functional dimensions, such as gelatinization behavior and water-holding capacity of Kodo millet, facilitate its use across diverse food formats, such as biscuits, porridges, and extruded snacks (18). However, fortification with legume flours can enhance protein density and amino acid score of such products, making them suitable for large-scale institutional feeding programs and household consumption alike. Empirical trials have shown that blending 70% Kodo millet flour with 30% soy or chickpea flour can increase the protein content of baked goods from 5.09% to over 11.8%, while also improving sensory acceptability (19).

Therefore, the present study is conceptualized as a novel intervention strategy focused on formulating and evaluating complementary millet-legume-based food products with an emphasis on lysine fortification. This study addresses a critical nutritional deficiency not just by increasing protein intake but by enhancing its biological utilization, having direct implications in child growth, cognitive development, and long-term health. By harnessing the advantages of traditional grains and legumes through modern processing and scientific evaluation, this research contributes toward a sustainable, scalable, and nutritionally sound approach to combat protein quality deficiency in resource-constrained populations.

## 2 Materials and methods

### 2.1 Procurement and processing of sample

Kodo millet (IPs 178), wheat (PBW 826), and rice (PR 131) were sourced from the Department of Plant Breeding and Genetics at Punjab Agricultural University (PAU), Ludhiana, immediately after harvest, and chickpea was purchased from the local market. The procured grains were cleaned, dried in a hot air oven (Model MSW 211, Macro Scientific Works Pvt. Ltd., India) to achieve desired moisture level (5%) and then finely ground into flour using high speed domestic stoneless flour mill (Saffron, Relitech Industries Pvt., Ltd., Ahmedabad, India) for subsequent nutritional analysis of the raw material.

### 2.2 Development of Kodo millet–chickpea–based food products

For the development of value-added products, a combination of Kodo millet (IPs 178) flour along with chickpea flour was used (Table 1). Chickpea has good-quality protein; therefore, it was selected for the development of products. The developed products were subsequently compared with recipes prepared using flours of cereal counterparts, such as wheat or rice, serving as controls. In the current study, the target group was school-going children, keeping in view their food preferences, millet-pulse-based products were developed. Offering milk as a meal or snack is a common dietary practice followed by parents for their school-going children. So, nutrimilletvita, which could be mixed with milk, was developed using Kodo millet flour and chickpea flour. Further, eating fried snacks in between meals is also common among children in India. Therefore, to fulfil the energy and protein requirements of this age, along with improvement in protein quality, a fried snack named crispy crunch was also developed.

**Table 1:** Development of Kodo millet-chickpea products.

Ingredients	Nutrivita		Crispy crunch	
	Control nutrivita	Kodo millet- chickpea nutrimilletvita	Control crispy crunch	Kodo millet-chickpea crispy crunch
Wheat (flour)	100%	–	20%	–
Rice flour	–	–	70%	20%
Kodo millet (flour)	–	50%	–	50%
Chickpea (flour)	–	50%	10%	30%
Milk powder	80%	80%	–	–
Cocoa powder	35%	35%	–	–
Cashew nut	25%	25%	–	–
Sugar	50%	80%	–	–
Salt	–	–	2.84%	2.84%
Butter	–	–	1tbsp	1tbsp
Baking soda	–	–	1.25%	1.25%
Corn flour	–	–	10%	10%
Methodology	Wheat grains were heated in a pan and roasted for 10 min, while cashew nuts were roasted for 6 min. The roasted wheat grains and cashew nuts were ground into a fine flour consistency. The wheat–cashew nut flour with cocoa powder, powdered sugar, and milk powder was combined until a homogeneous fine powder was obtained. For millet preparation, the millet was soaked until sprouting occurred. Following sprouting, Kodo millet, chickpeas, and cashew nuts were individually roasted for 10 min, and the roasted millet, chickpeas, and cashew nuts were ground into a fine powder. The millet-chickpea-cashew nut flour with cocoa powder, powdered sugar, and milk powder was mixed to create a comprehensive powdered mixture (20)		In a bowl, plain flour, baking soda, and salt were combined, ensuring thorough mixing, and water was added and whisked until a slightly thick and smooth batter was achieved. In a pan, 1 tablespoon of butter was melted and evenly spread. The batter was poured into the pan and stirred continuously over low heat until it begins to hold its shape. The batter was allowed to cool, then incorporated with 1 tablespoon of corn flour to form a cohesive dough. The dough was shaped into small round balls and loaded them into a *chakli* maker. The dough was extruded into longitudinal shapes. Oil was heated and the shaped raw crispy crunches were added and deep-fried until golden brown and crispy (20).	

### 2.3 Organoleptic evaluation of the developed food products

**Figure 1:**
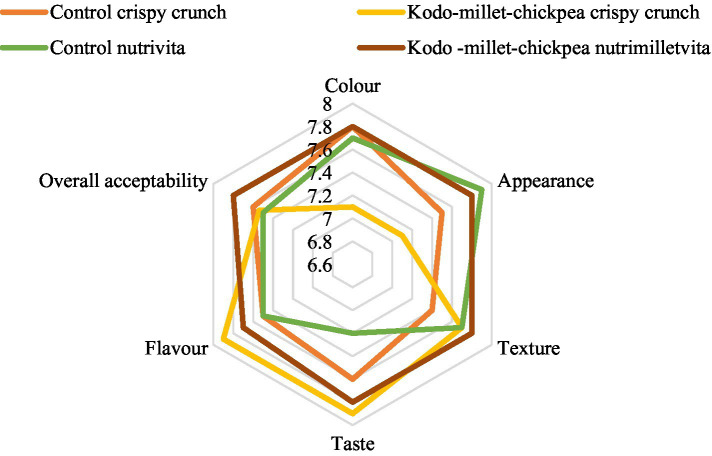
Organoleptic evaluation of Kodo millet–chickpea products.

Semi-trained individuals affiliated with the Department of Food and Nutrition at Punjab Agricultural University, Ludhiana, conducted sensory evaluations of standardized products derived from Kodo millet–chickpea combination, which were assessed on the basis of various sensory attributes, such as color, appearance, texture, taste, flavor, and overall acceptability (Figure 1). The evaluation was performed utilizing a nine-point Hedonic rating scale, where a rating of 9 represented “like extremely” and a rating of 1 represented “dislike extremely” (21).

### 2.4 Nutritional evaluation of millet pulse-based food products

The developed products derived from Kodo millet–chickpea, alongside their respective control counterparts, prepared from conventional cereal sources such as wheat or rice, underwent comprehensive analysis for various nutritional parameters such as proximate composition, estimation of amino acid content, estimation of protein digestibility, and protein digestibility corrected amino acid score (PDCAAS).

#### 2.4.1 Proximate composition

Moisture, crude protein, crude fat, crude fiber, and ash were analyzed using three replications of each formulation (22). A 5 g sample was weighed and placed in three pre-weighed China crucibles in a hot air oven at 105°C for 8 h to dry to a constant weight to determine its moisture content. Nitrogen determination was done using the macro Kjeldahl method (KEL PLUS KES 12 L RTS, Pelican Equipments, India), where nitrogen was converted to crude protein using a conversion factor of 6.25. The crude fat content was determined using the Automatic Soxhlet apparatus (SOCSPLUS—SCS 06 AS DLS TS), moisture-free sample was placed in a thimble, and fat was extracted using petroleum ether as a solvent. The extracted fat from the sample was weighed after evaporating the remaining solvent. Crude fiber was determined using a 5 g (moisture and fat-free) sample by refluxing with 1.25% sulfuric acid initially, followed by 1.25% sodium hydroxide (NaOH). It was further oven-dried, and ash content was analyzed by igniting the weighed samples at 550 °C in a muffle furnace (Laboratory Model NSW 101, Narang Scientific Works Pvt., Ltd., India) for 4 h. After cooling in a desiccator, the crucible with residue was weighed again. Total carbohydrates were calculated by subtracting the sum of all proximate parameters (moisture content, crude protein, crude fat, crude fiber, and total ash) from 100.

#### 2.4.2 Amino acids by GC–MS reagents amino acid extraction

The sample (0.2 g) was homogenized with 1.5 mL of 0.1 M HCl and centrifuged (Abdos DMO412). The supernatant was collected and stored (−80°C) until analysis. For the deproteinization and derivatization, the extract (100 μL) was combined with 250 μL of acetonitrile in a safe-lock micro test tube and centrifuged (10,000 rpm/3 min). The supernatant was mixed with 100 μL of an internal standard (IS) solution (5 μg/mL) and transferred to heat-resistant tubes, subsequently dried under nitrogen. 1-Chloromethane (50 μL) was added to the dried samples and the mixture was evaporated. Further, MTBSTFA (50 μL) and acetonitrile (50 μL) were added and incubated (100°C for 60 min), followed by refrigeration for storage and later analyzed by GC–MS (GCMS 2400 System, PerkinElmer, United States) for amino acids.

#### 2.4.3 *In vitro* protein digestibility

*In vitro* protein digestibility was analyzed by mixing dry sample (0.5 g) with pepsin solution (50 mL) in a 250 mL conical flask, followed by incubation at 37°C for 24 h (Waiometra OSIV-5642). The solution was then neutralized with approximately 30 mL of 0.2 N NaOH, after which pancreatin solution (50 mL) was added and again incubated (37°C for 24 h). An enzyme blank (without sample) was concurrently run under prescribed conditions, and an aseptic environment in the system was maintained by adding a few drops of toluene. Subsequently, the contents were centrifuged at high speed and filtered through Whatman No. 44 filter paper, and the residue was analyzed for nitrogen content using the macro-Kjeldahl method. The digestibility coefficient was determined by subtracting the residual protein from the initial protein content based on 100 g of the sample (23).

#### 2.4.4 Protein digestibility corrected amino acid score (24)

PDCAAS was calculated using the following formula.

PDCAAS = (mg of limiting amino acid in 1 g of test protein/mg of the same amino acid in 1 g reference protein) × *in vitro* protein digestibility,

where lysine was identified as the most limiting amino acid. The reference amino acid pattern corresponded to the amino acid requirements of preschool children aged 2–5 years, and values exceeding 1.00 were adjusted and recorded as 1.00.

### 2.5 Shelf-life evaluation of the developed food products

Developed food products were kept in low-density polyethylene pouches (thickness: 51 microns) at room temperature. The organoleptic characteristics of the stored products were evaluated every 15 days using a 9-point hedonic scale for 3 months (25).

### 2.6 Consumer acceptability of the developed food products

**Figure 2:**
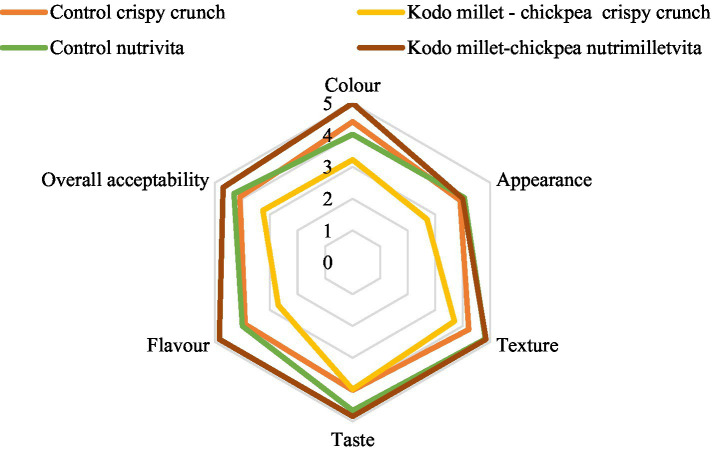
Consumer acceptability of Kodo millet–chickpea crispy crunch and nutrimilletvita (100 g, dry weight basis).

Consumer acceptability of the developed products was assessed by at least 50 school-going children (7–12 years) using a Likert Rating Scale. It provides a quantitative way to capture respondents’ attitudes and perceptions on a scale ranging from extremely liked to extremely disliked with scores of 5 and 1, respectively (Figure 2).

### 2.7 Statistical analysis

**Figure 3:**
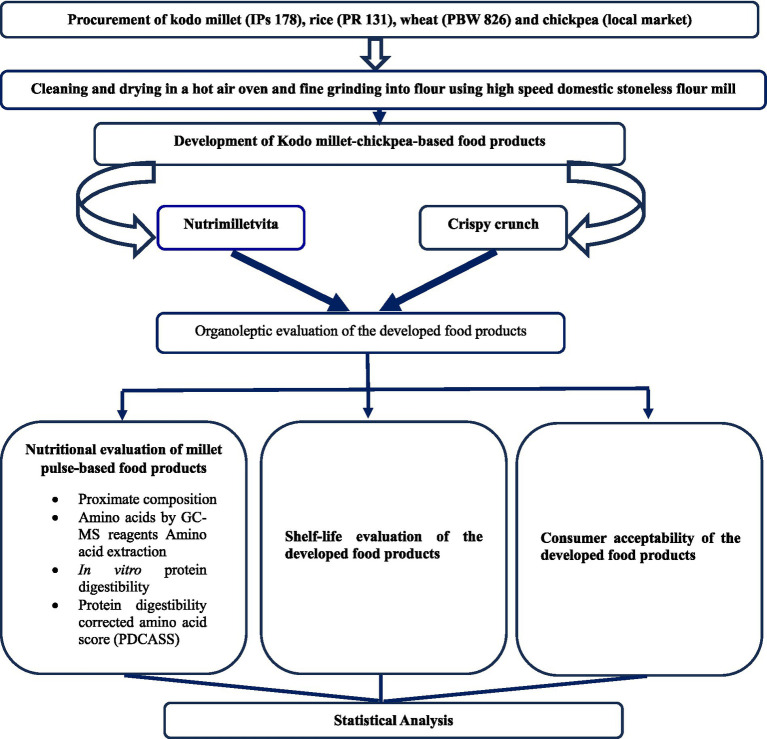
Methodology of the study.

The data were analyzed in a completely randomized design using SPSS software (26 version). Mean and standard deviation for the various parameters were computed. Analysis of Variance (One-way ANOVA), Kruskal-Wallis test, and Mann–Whitney test were employed to assess the sensory and nutritional parameters of developed value-added food products prepared from Kodo millet–chickpea. The methodology of the study has been illustrated in Figure 3.

## 3 Results

### 3.1 Organoleptic evaluation of Kodo millet–chickpea products

The developed foods were subjected to organoleptic evaluation by a panel of semi-trained judges, and the results revealed that among the developed products, nutrimilletvita obtained a higher overall acceptability score (7.80 ± 0.42) than crispy crunch (7.54 ± 0.34) (Figure 1). Both the developed products received comparable overall acceptability scores with respect to their control counterparts and were found to be acceptable by the panel of judges by obtaining a score of more than 7.5. A significant difference (*p* ≤ 0.01) in the sensory characteristics (color, texture, taste, and flavor) was found between the prepared crispy crunch and its control, while nutrimilletvita displayed a non-significant difference.

### 3.2 Nutritional composition

#### 3.2.1 Proximate composition of Kodo millet–chickpea products

The crude protein content in the Kodo millet–chickpea–based value-added products ranged from 6.90 ± 0.00 to 18.31 ± 2.85 g/100 g on a dry weight basis, with a higher content in Kodo millet–chickpea nutrimilletvita. The observed protein content in developed products was significantly (*p* ≤ 0.01) higher than their respective control counterparts (Table 2). Significantly (*p* ≤ 0.01) higher crude fiber content was also observed in Kodo millet–chickpea nutrimilletvita (5.00 ± 0.60 g/100 g) as compared with Kodo millet–chickpea crispy crunch, where both the developed products displayed significantly (*p* ≤ 0.01) higher crude fiber content than their corresponding control products. However, the crude fat content varied significantly (*p* ≤ 0.01) in the developed products and ranged from 7.80 ± 0.26 to 25.60 ± 1.15 g/100 g, with maximum content exhibited by Kodo millet–chickpea crispy crunch.

**Table 2:** Nutritional composition of Kodo millet–chickpea products (g/100 g, dry weight basis).

Products	Moisture (%)	Crude protein (%)	Crude fat (%)	Crude fiber (%)	Ash (%)	CHO (%)
Control crispy crunch	5.80 ± 0.55	06.90 ± 0.00	17.03 ± 0.64	0.53 ± 0.51	0.11 ± 0.00	69.63 ± 0.00
Kodo millet-chickpea crispy crunch	5.76 ± 1.02^**^	10.01 ± 0.25^**^	25.60 ± 1.15^**^	3.38 ± 0.34^**^	1.38 ± 0.56^**^	53.88 ± 0.137**
Control nutrivita	9.46 ± 0.73	16.00 ± 2.00	07.80 ± 0.26	1.66 ± 0.61	1.40 ± 0.51	63.68 ± 2.03
Kodo millet-chickpea nutrimilletvita	8.76 ± 0.70^NS^	18.31 ± 2.85^**^	08.10 ± 0.26^**^	5.00 ± 0.60^NS^	1.94 ± 0.66^NS^	57.89 ± 0.93^**^

Values are expressed as Mean ± SD of triplicates. Values in columns having control and experimental groups only show **significance at (*p* ≤ 0.01) level.

#### 3.2.2 Amino acid composition

The indispensable amino acid profile of the products prepared from Kodo millet–chickpea revealed that the amount of the most limiting amino acid, lysine, in cereal diets was found to be significantly (*p* ≤ 0.01) higher in the developed products than their control counterparts (Table 3). Significantly (*p* ≤ 0.01) higher lysine content was observed in Kodo millet–chickpea crispy crunch (4.06 ± 0.56 g/100 g). Other indispensable amino acids, such as, methionine, valine, and leucine varied in a narrow range. Kodo millet–chickpea crispy crunch was found to have higher values of many indispensable amino acids to the tune of leucine (16.02 ± 0.03 g/100 g), phenylalanine (6.71 ± 0.16 g/100 g), isoleucine (7.02 ± 0.10 g/100 g), and tryptophan (2.06 ± 0.02 g/100 g). Similarly, significantly (*p* ≤ 0.01) higher leucine (15.03 ± 0.74 g/100 g) and isoleucine (5.19 ± 0.09 g/100 g) content was displayed by the developed nutrimilletvita than its control counterpart.

**Table 3:** Amino acid composition of Kodo millet–chickpea products (g/100 g, dry weight basis).

Indispensable amino acids									
Products	Leu	Lys	Met	Phen	Iso	Val	Thr	Trp	His
Control crispy crunch	08.28 ± 0.20	3.50 ± 0.75	1.49 ± 0.03	3.73 ± 0.07	3.17 ± 0.08	2.14 ± 0.03	1.52 ± 0.20	1.06 ± 0.04	1.73 ± 0.02
Kodo millet-chickpea crispy crunch	16.02 ± 0.03^**^	4.06 ± 0.56^**^	1.10 ± 0.03^NS^	6.71 ± 0.16^**^	7.02 ± 0.10^**^	4.71 ± 0.09^**^	2.45 ± 0.25^**^	2.06 ± 0.02^**^	0.65 ± 0.28^**^
Control nutrivita	07.83 ± 0.14	3.98 ± 0.46	1.52 ± 0.01	2.85 ± 0.06	3.21 ± 0.04	2.07 ± 0.02	1.70 ± 0.01	1.47 ± 0.44	1.20 ± 0.02
Kodo millet-chickpea nutrimilletvita	15.03 ± 0.74^**^	3.96 ± 0.06^NS^	1.51 ± 0.03^NS^	5.76 ± 0.02^NS^	5.19 ± 0.09^**^	6.31 ± 0.02^**^	3.74 ± 0.02^NS^	1.51 ± 0.21^NS^	0.95 ± 0.14^NS^

Dispensable amino acids									
Products	Ala	Arg	Asp	Cys	Glu	Gly	Pro	Ser	Tyr
Control crispy crunch	7.35 ± 0.29	2.70 ± 0.01	4.50 ± 0.10	1.65 ± 0.04	23.53 ± 1.84	6.40 ± 0.09	9.30 ± 0.20	4.70 ± 0.10	3.94 ± 0.05
Kodo millet-chickpea crispy crunch	9.04 ± 0.02^**^	0.88 ± 0.10^**^	7.06 ± 0.15^**^	0.57 ± 0.10^**^	22.36 ± 0.41^**^	3.28 ± 0.41**	7.24 ± 0.26^**^	4.25 ± 0.14^NS^	1.51 ± 0.01^**^
Control nutrivita	7.16 ± 0.05	2.56 ± 0.27	4.68 ± 0.18	1.75 ± 0.11	34.14 ± 1.37	6.51 ± 0.23	9.48 ± 0.27	4.37 ± 0.03	3.72 ± 0.04
Kodo millet-chickpea nutrimilletvita	3.35 ± 4.06^NS^	0.81 ± 0.22^NS^	7.66 ± 0.11^*^	1.01 ± 0.32^NS^	22.37 ± 1.11^**^	2.39 ± 0.33^NS^	8.38 ± 0.40^NS^	4.60 ± 0.17^NS^	1.75 ± 0.11

Leu-leucine, Lys-lysine, Met-Methionine, Phe-phenylalanine, Iso-Isoleucine, Val-Valine, Thr-Threonine, His-Histdine, Ala-Alanine, Arg-Arginine, Asp-Aspartic acid, Cys-Cysteine, Glu-Glutamic acid Gly-Glycine. Pro-Proline, Ser-Serine and Tyrosine Tyr, C-Control, E-Finger Millet-Chickpea. Values are expressed as Mean ± SD of triplicates. Values in columns having control and experimental groups only show **significance at (*p* ≤ 0.01) level.

However, the data about dispensable amino acids of the developed products revealed that all the dispensable amino acids, such as alanine, arginine, aspartic acid, cysteine, glutamic acid, glycine, proline, serine, and tyrosine, varied significantly (*p* ≤ 0.01) among the developed products. However, the values of tyrosine were found to be lower in all the products in comparison to other amino acids.

#### 3.2.3 *In vitro* protein digestibility and PDCAAS of the products prepared from Kodo millet–chickpea

**Figure 4:**
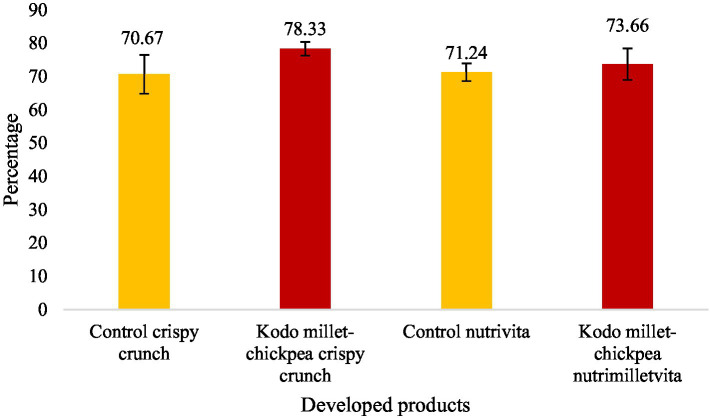
*In vitro* protein digestibility of Kodo millet–chickpea products (100 g, dry weight basis).

**Figure 5:**
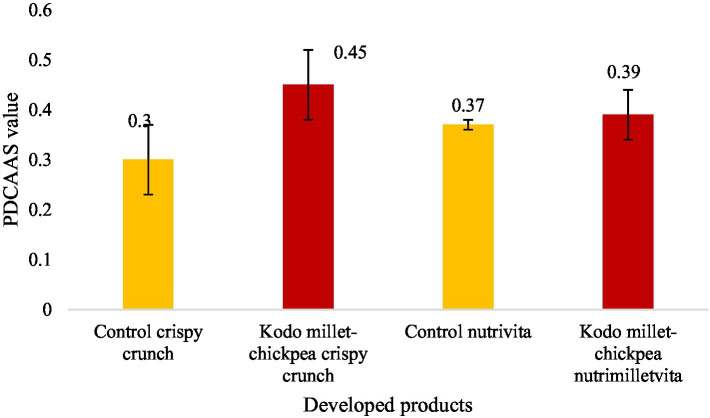
Protein digestibility corrected amino acid score (PDCAAS) of Kodo millet–chickpea products (100 g, dry weight basis).

In the current investigation, the *in vitro* protein digestibility (IVPD) and PDCAAS of the developed nutrimilletvita were found to be 73.66 ± 5.86 percent and 0.39 ± 0.07, respectively, which was significantly (*p* ≤ 0.01) lower than 78.33 ± 2.08 percent and 0.45 ± 0.07, respectively, in Kodo millet–chickpea crispy crunch (Figures 4, 5). However, significantly (*p* ≤ 0.01) lower *in vitro* protein digestibility and PDCAAS values were also observed in the respective control groups of the developed products.

### 3.3 Consumer acceptability of the products prepared from Kodo millet-chickpea

The developed products were tested for their acceptability by the consumers (Figure 2). Fifty children in the age group of 7–12 years were asked to evaluate the developed products based on the Likert scale. Both the developed products were found to attain an overall acceptability score of more than 3, with a higher score displayed by Kodo millet–chickpea nutrimilletvita (4.70 ± 0.21). The scores obtained were found to be statistically significant (*p* ≤ 0.01) when analyzed using the Kruskal–Wallis test and Mann–Whitney test.

### 3.4 Shelf-life evaluation of the products prepared from Kodo millet-chickpea

The food products developed from a combination of Kodo millet and chickpea, were sealed in low-density polyethylene pouches (thickness-51 microns) and stored at a constant room temperature (37 °C) in cool and dry conditions, which were evaluated every 15 days over 3 months through sensory assessments conducted by a semi-trained panel using a 9-point Hedonic scale (Tables 4, 5). The sensory scores of the developed products, Crispy Crunch and Nutrimilletvita, were 7.90 ± 0.52 and 8.10 ± 0.52, respectively, on the 0th day. These scores gradually declined to 5.45 ± 0.57 and 5.69 ± 0.34 by the 90th day, showing a significant (*p* ≤ 0.01) decrease over the 3-month storage period.

**Table 4:** Sensory scores of crispy crunch prepared from kodo millet-chickpea at an interval of 15 days for 3 months.

Products	Color	Appearance	Texture	Taste	Flavor	Overall acceptability
0th Day						
Control crispy crunch	7.80 ± 0.42	7.50 ± 0.53	7.40 ± 0.52	7.60 ± 0.52	7.50 ± 0.53	7.70 ± 0.52
Kodo millet–chickpea crispy crunch	6.67 ± 0.55^**^	6.80 ± 0.52^**^	7.50 ± 0.53^**^	8.00 ± 0.70^**^	7.40 ± 0.84^**^	7.90 ± 0.52^**^
15th day						
Control crispy crunch	7.80 ± 0.42	7.50 ± 0.53	7.40 ± 0.52	7.50 ± 0.53	7.50 ± 0.52	7.70 ± 0.52
Kodo millet–chickpea crispy crunch	6.67 ± 0.55^**^	6.80 ± 0.52^**^	7.50 ± 0.53^**^	8.00 ± 0.84^**^	7.40 ± 0.84^**^	7.90 ± 0.52^**^
30th day						
Control crispy crunch	7.80 ± 0.42	7.40 ± 0.52	7.30 ± 0.48	7.30 ± 0.52	7.50 ± 0.53	7.70 ± 0.52
Kodo millet–chickpea crispy crunch	6.67 ± 0.55^**^	6.60 ± 0.70^**^	7.40 ± 0.52^**^	7.70 ± 0.43^**^	7.40 ± 0.70^**^	7.80 ± 0.75^**^
45th day						
Control crispy crunch	7.60 ± 0.52	7.40 ± 0.52	7.20 ± 0.34	7.30 ± 0.48	7.20 ± 0.42	7.30 ± 0.47
Kodo millet–chickpea crispy crunch	6.64 ± 0.45^**^	6.65 ± 0.52^**^	6.80 ± 0.56^**^	7.29 ± 0.42^**^	7.30 ± 0.48^**^	7.20 ± 0.32^**^
60th day						
Control crispy crunch	7.60 ± 0.52	7.40 ± 0.52	7.20 ± 0.34	6.90 ± 0.48	7.20 ± 0.42	7.20 ± 0.47
Kodo millet–chickpea crispy crunch	6.64 ± 0.45^**^	6.65 ± 0.52^**^	6.30 ± 0.56^**^	6.29 ± 0.42^**^	7.30 ± 0.48^**^	6.60 ± 0.32^**^
75th Day						
Control crispy crunch	6.60 ± 0.56	6.40 ± 0.42	6.20 ± 0.34	6.30 ± 0.48	6.23 ± 0.45	6.70 ± 0.53
Kodo millet–chickpea crispy crunch	6.34 ± 0.47^**^	6.34 ± 0.55^**^	6.30 ± 0.56^**^	5.29 ± 0.42^**^	5.30 ± 0.50^**^	5.94 ± 0.37^**^
90th day						
Control crispy crunch	6.40 ± 0.59	5.80 ± 0. 57	5.29 ± 0.44	5.89 ± 0.66	6.00 ± 0.65	6.00 ± 0.63
Kodo millet–chickpea crispy crunch	6.24 ± 0.66^**^	6.14 ± 0.68^**^	5.37 ± 0.56^**^	5.00 ± 0.52^**^	5.00 ± 0.60^**^	5.45 ± 0.57^**^

Values are expressed as Mean ± SD of triplicates.

** Values are significant at 1% level of significance.

**Table 5:** Sensory scores of nutrimilletvita prepared from kodo millet–chickpea at an interval of 15 days for 3 months.

Products	Color	Appearance	Texture	Taste	Flavor	Overall acceptability
0th day						
Control nutrivita	7.70 ± 0.48	7.70 ± 0.45	7.70 ± 0.42	7.70 ± 0.67	7.60 ± 0.61	7.70 ± 0.61
Kodo millet–chickpea nutrimilletvita	7.80 ± 0.63	8.80 ± 0.53^NS^	7.80 ± 0.53^NS^	8.80 ± 0.42^NS^	7.99 ± 0.69^NS^	8.10 ± 0.52^NS^
15th day						
Control nutrivita	7.70 ± 0.48	7.70 ± 0.45	7.70 ± 0.42	7.70 ± 0.67	7.60 ± 0.61	7.55 ± 0.61
Kodo millet–chickpea nutrimilletvita	7.80 ± 0.63^NS^	7.80 ± 0.53^NS^	7.80 ± 0.53^NS^	7.80 ± 0.42^NS^	7.89 ± 0.69^NS^	7.90 ± 0.52^NS^
30th day						
Control nutrivita	7.60 ± 0.58	7.68 ± 0.48	7.60 ± 0.52	7.60 ± 0.70	7.50 ± 0.71	7.59 ± 0.61
Kodo millet–chickpea nutrimilletvita	7.69 ± 0.62^NS^	7.70 ± 0.82	7.70 ± 0.63	7.70 ± 0.48^NS^	7.70 ± 0.80^NS^	7.60 ± 0.72
45th day						
Control nutrivita	7.40 ± 0.52	7.30 ± 0.48	5.30 ± 0.48	6.40 ± 0.48	6.30 ± 0.67	6.20 ± 0.42
Kodo millet–chickpea nutrimilletvita	7.50 ± 0.70	7.40 ± 0.70^*^	7.42 ± 0.63	6.50 ± 0.42^**^	6.40 ± 0.52	7.20 ± 0.71^**^
60th day						
Control nutrivita	6.20 ± 0.67	7.10 ± 0.57	7.20 ± 0.42	7.30 ± 0.44	7.00 ± 0.60	6.00 ± 0.54
Kodo millet–chickpea nutrimilletvita	6.40 ± 0.52	7.30 ± 0.48^*^	7.39 ± 0.50^**^	7.42 ± 0.55^*^	7.32 ± 0.49	6.98 ± 0.56^**^
75th day						
Control nutrivita	6.00 ± 0.57	6.99 ± 0.47	6.00 ± 0.48	6.04 ± 0.38	5.60 ± 0.50	5.60 ± 0.38
Kodo millet–chickpea nutrimilletvita	6.20 ± 0.50	7.00 ± 0.38^**^	6.89 ± 0.52^*^	6.16 ± 0.46	6.86 ± 0.49^*^	6.00 ± 0.45^*^
90th day						
Control nutrivita	5.99 ± 0.43	6.30 ± 0.45	4.00 ± 0.35	4.56 ± 0.38	4.80 ± 0.53	4.94 ± 0.48
Kodo millet–chickpea nutrimilletvita	6.10 ± 0.55	6.52 ± 0.39^*^	5.23 ± 0.38	4.86 ± 0.46^NS^	5.99 ± 0.58^*^	5.69 ± 0.34^NS^

Values are expressed as Mean ± SD of triplicates.

**Values are significant at 1% level of significance.

### 3.5 Nutritional composition of the products prepared from Kodo millet–chickpea (on a per-serving basis)

**Figure 6:**
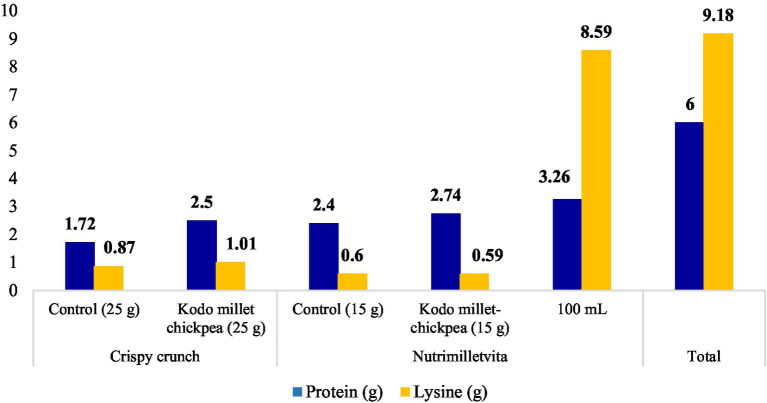
Nutritional composition of Kodo millet-chickpea product (per serving basis).

The portion size of the developed products was calculated using the standardized measuring containers. The serving size of crispy crunch was standardized as 25 g and nutrimilletvita as 15 g. The nutritional composition of the products prepared from Kodo millet–chickpea based was worked out. The developed Kodo millet–chickpea-based crispy crunch provided 2.5 g of protein and 1.01 g of lysine, whereas the prepared nutrimilletvita, which should be taken along with milk (100 mL), provided 6 g of protein and 9.18 g of lysine (Figure 6). Therefore, consuming one serving of prepared products was found to meet about 22–41% of the recommended dietary allowance (RDA) for protein and fulfilled approximately 100% of the lysine requirement (The RDAs mentioned in the RDA table by ICMR for 13–15-year-old children have been considered for calculating the percent contribution of the product in meeting RDA).

### 3.6 Cost estimation of the products prepared from Kodo millet-chickpea

The cost estimation of the product was done considering the raw ingredients used along with overhead charges (30%) on a per-serving basis. It was observed that the crispy crunch was prepared with the amount of Rs. 7.80 (on a per-serving basis), while nutrimilletvita was prepared with a cost of Rs. 27.15, with a milk-inclusive cost of Rs. 33.15/−.

### 3.7 Cost–benefit ratio of finger millet-chickpea product

The cost–benefit ratio of crispy crunch and nutrimilletvita prepared from Kodo millet–chickpea combination was found to be 1.42 and 1.17, respectively, which shows that the developed products are profitable and sustainable.

## 4 Discussion

### 4.1 Organoleptic evaluation of Kodo millet-chickpea products

Organoleptic properties of a food are perceived by human senses, such as smell, touch, sight, and taste. The acceptance or rejection of food is predicted solely by its ability to meet consumer expectations and needs (26). In the current study, cereals were replaced with a combination of millets and chickpeas to develop a high-quality protein product optimized to meet the preferences of school-going children. Kodo millet was replaced with wheat and rice from 30 to 50 percent, depending upon the acceptability of the products. As crispy crunch is preferred and liked by the targeted group, it obtained an overall acceptability score of more than 7.5.

However, the crispy crunch prepared from Kodo millet–chickpea obtained a score lower than the control, primarily due to the fibrous residue left after chewing (27), attributable to the high fiber content of Kodo millet, which also proved to be the highest among all flours used in the preparation of the products However, the Kodo millet–chickpea crispy crunch prepared in the present investigation was characterized by its darker hue and inconsistent texture and received lower scores in both color and overall acceptability compared with its control counterparts. Similar results were reported by a research study that revealed an overall acceptability score ranging from 7.65 to 8.25 in Kurkure developed from Kodo millet (28). Also, the nutrimilletvita developed from Kodo millet–chickpea was in powdered form and had to be consumed with milk and had higher scores than the control. A similar study reported that the preparation of a weaning food using millet and pulse combination reported higher acceptability in nutritional and sensory quality compared to its counterparts (29). These findings were substantiated through an analysis of various physical, chemical, and sensory attributes. Overall, among both the developed products, Kodo millet–chickpea nutrimilletvita obtained a higher overall acceptability score.

### 4.2 Proximate composition of Kodo millet–chickpea products

In the current study, the crude protein content was found to be more than 10 g/100 g in both the developed products, which could be attributed to the millet and pulse combination. The protein present in millets is higher than in traditional cereal wheat and rice, out of which 44.7% of the amino acids are essential (30). The nutrimilletvita had higher protein content than crispy crunch, which could be due to the use of a higher amount of millet and pulse used in the preparation of nutrimilletvita. Moreover, deep frying of crispy crunch might have led to its lower protein content. A similar trend of protein in fried snacks made up of Kodo millet, having 11.08 percent protein content, was reported (31). It was observed that the developed value-added food products had higher protein content than traditional counterparts, indicating that these can be used as a nutrient-dense food by the targeted group and may help in alleviating protein deficiency among them. Moreover, the developed products are mostly preferred by children, so there are chances of more consumption of these products, leading to their higher nutritional status. The results of the current investigation are in line with other research findings, which reported protein content of 12.25 g/100 g in developed nutri-rich RTE using finger millet and corn in the ratio of 25:75 (32).

The variation in the crude fat content among the different products can be attributed to the use of different ingredients for their preparation. Moreover, it was observed that Kodo millet–chickpea crispy crunch had higher fat content during frying as compared with other products. Both the products developed from Kodo millet in the current study had higher fat content than their control counterparts, which can be due to the greater fat absorption by the millet as it has higher fiber content (7.40 ± 0.39 g/100 g). Moreover, Kodo millet has high amylose content, which forms a porous structure and swells when cooked, allowing more fat to absorb (33, 34). A similar range of fat content in millet-based products was found in finger millet chocolate (23.62 g/100 g), porso millet *chakli* (30.47 g/100 g), finger millet cookies (15 g/100 g), and instant health mix (35–37).

The crude fiber content of any product depends upon the processing techniques used for its preparation. In the current study, the Kodo millet–based products were found to have a higher crude fiber content, which can be attributed to more fiber present in the grain (7.40 ± 0.39), as compared with the other grains used in the study. The results showed that the fiber content was higher in all the developed products than their control counterparts, which can be due to the presence of higher amounts of fiber in millet and pulse grains. Similarly, other research evidence reported crude fiber content of 5.09 and 7.86 g/100 g in Kodo millet *mathri* and chocofills (32, 38).

### 4.3 Amino acid composition

The presence of indispensable amino acids in a food improves its quality, and the absence of any one of them makes the protein inferior, and it is not utilized by the body to make muscle protein (39). Hence, a food must have all the indispensable amino acids. In India, cereal-based diets offer poor quality protein with quality protein differences ranging from 4 to 26 percent (40). Therefore, in the current study, an attempt was made to replace cereals with millets and pulses to improve the quantity and quality of protein.

The food products developed with a millet-pulse combination resulted in a better amino acid profile. Lysine, the limiting amino acid of cereals, was found to be increased in the developed products. The maximum lysine percent increase content was observed in Kodo millet–chickpea crispy crunch (16.0%). The increase in lysine content of the developed products can be attributed to the amino acid profile of millets and pulses, complementary amino acid combination, and their synergetic effect. It was reported that millet pulse blend provided a balanced amino acid profile, particularly increased lysine levels (28).

The data obtained from analysis of dispensable amino acids showed that all of them, such as alanine, arginine, aspartic acid, cysteine, glutamic acid, glycine, proline, serine, and tyrosine, are present in sufficient amounts in the developed products. It is believed that these can be synthesized by the body and so are not required in the diet. However, the presence of dispensable amino acids in food is also important as they help to balance the overall amino acid profile of food. This balance is important for efficient protein synthesis and utilization in the body. Moreover, foods that contain a variety of amino acids, such as dispensable amino acids, contribute to a more balanced protein source (41). An investigation on the dispensable amino acid profile of millet pulse-based products found that dispensable amino acids, such as arginine, aspartic acid, glutamic acid, and glycine content, increased in the products than their control counterparts (42).

### 4.4 *In vitro* protein digestibility and PDCAAS

*In vitro* protein digestibility of any product depends upon its amino acid profile, protein content, pH ionic strength, temperature, and anti-nutritional factors. The processing technology applied also affects the digestibility. The *in vitro* protein digestibility of the developed products was found to be more than 70%,% with a digestibility of 78.33% in Kodo millet–chickpea crispy crunch. The higher protein digestibility depicts the balanced amino acid profile in the ingredients used for the development of the products. The *in vitro* protein digestibility led to a high PDCAAS value of more than 0.4 in Kodo millet–chickpea crispy crunch (0.45). The combination of millet and pulse has led to the improvement in protein digestibility and PDCAAS since this combination makes a more balanced amino acid profile, improving overall protein quality (43). Similar results in the *in vitro* protein digestibility and protein digestibility–corrected amino acid score of the millet soybean blend had been observed (44). From the foregoing results, it can be interpreted that millet pulse–based foods can help in improving nutritional health and food security, especially in developing countries, where cereals are consumed as staple foods. The consumption of these newly developed foods by the vulnerable population may improve their nutritional status, decreasing the prevalence of undernutrition.

### 4.5 Consumer acceptability of Kodo millet–chickpea products

Consumer acceptability evaluation scale is a measure of using the Likert scale to determine the liking of consumers for food items in terms of their taste, texture, appearance, flavor, etc. In the current study, the developed products obtained scores comparable with their control counterparts, depicting the consumer’s acceptability toward this product. The low score of Kodo millet–chickpea can be due to the dark green color that the product attained after frying, which is clear from the scores for color (3.22), appearance (2.70), texture (3.70), taste (4.00), and flavor (2.70). Moreover, the product observed had higher fat and was commented as oily due to more fat absorption by the millet as it has higher fiber content (7.40 ± 0.39 g/100 g). Moreover, Kodo millet has high amylose content, which forms a porous structure and swells when cooked, allowing more fat to be absorbed. On the contrary, the developed nutrimilletvita obtained a significantly higher overall acceptability score of 4.70 as compared with the developed crispy crunch as well as the control nutrivita. This could be attributed to the addition of milk powder and cocoa powder along with millet powder, making an acceptable combination for consumption, particularly by school-going children. The consumers showed great satisfaction and liking for the developed Kodo millet–chickpea–based products, which is clear from the overall acceptability score. Evidence suggested that pearl millet-based *dalia* received higher acceptability scores compared with their control counterpart (45). Contrary to the present study, a study reported the reception of low organoleptic scores (3.1 to 3.7) of finger millet-based food products (46). The addition of chickpeas to the products in the current study might have improved the taste and overall consumer acceptability. The combination not only balances the flavor profile but also improves texture and nutritional quality, making the products more appealing and beneficial. Likewise, the addition of pulses was reported to improve the unique aroma and texture of millet-based foods (47, 48).

### 4.6 Shelf-life evaluation of Kodo millet–chickpea products

Millets with high-fat content can oxidize when exposed to air at varying rates, which degrades both their nutritional value and sensory quality. The shelf life of millet flour is shortened because of oxidative and hydrolytic deterioration (49). The lower shelf life of millet is mainly due to the presence of enzymes such as lipase and lipoxygenase, which trigger the oxidation of a large amount of fat present in flour, resulting in the production of an off-odor and flavor. As a result, rancidity is one of the most significant drawbacks of millet consumption (50). The oxidation of phenolic components by polyphenol oxidase and peroxidases, in addition to fatty acid oxidation, causes off-flavor and browning of millet flour during storage (51). To study the changes in organoleptic parameters of the developed products, the products were stored for 3-month periods and evaluated at an interval of 15 days. It was observed that the acceptability decreased with an increase in storage period. The developed products were acceptable for a period of 45 days, after which less acceptability scores were obtained. Similarly, a significant drop in the overall acceptability of a millet-based composite sports bar was noted from 38.3 to 13.1 over 3 months, while another report documented a comparable trend in karonda candy, whereby the overall acceptability gradually fell from 8.01 to 7.74 for unripe and from 7.78 to 6.98 for ripe candies over a 6-month storage period (52, 53).

### 4.7 Nutritional composition (per serving basis) of Kodo millet-chickpea products

The millet pulse-based product has been developed for school-going children, so it is important to know the amount of nutrients on a per serving basis. The developed food products were found to be rich in protein and lysine based on their one portion in comparison to their control counterparts. Hence, consumption of one portion of the millet pulse–based developed in the current study can help in combating protein malnutrition and improving protein quality in the country. Evidence emphasized that adding pulses to millet-based food increases both protein and lysine content (54).

### 4.8 Cost estimation of Kodo millet-chickpea food products

The cost of the developed product was compared with the available options in the market. It was observed that the same serving size of the crispy crunch of Brand A (25 g) is available at Rs. 24.87/−, while the developed product had a cost of Rs. 7.80/− Similarly, nutrimilletvita (100 g) was also found to be prepared at a lesser price of 11.21% per gram compared with varied options in the market. The data obtained highlighted the fact that the developed product involved less money in its preparation than its control and market counterparts. Moreover, these products had higher nutritional value. Therefore, the products developed in the current study are economical options for recommendation to the target group for combating undernutrition. Research demonstrated that the developed finger millet mix jaggery chocolate not only offers substantial cost savings but also boasts a superior nutrient profile when compared with a commercially available market product (35).

## 5 Conclusion

In the present investigation, the combination of Kodo millet and chickpea demonstrated an enhanced potential for the development of value-added, cost-effective products characterized by high organoleptic quality, enhanced nutritional attributes, extended shelf-life, and strong consumer acceptability. The formulated products delivered good-quality protein, exhibited improved *in vitro* protein digestibility, and achieved a higher PDCAAS. The products also demonstrated greater consumer acceptance and exhibited a shelf-life of up to 45 days. Based on these findings, it can be inferred that millet- and pulse-based foods have the potential to enhance nutritional health and promote food security, particularly in developing countries where cereals serve as staple foods. Thus, products developed from Kodo millet and chickpea demonstrated superior nutritional value per serving compared with conventional cereal-based foods, presenting an effective approach to addressing protein malnutrition and enhancing food security. Millet–pulse combinations are especially recommended for vulnerable populations, such as school-aged children, pregnant women, and adolescent girls, to ensure adequate intake of high-quality protein. These nutrient-dense products can also be integrated into National Feeding Programs to combat malnutrition at the community level. The potential of millet-based food products for children appears highly encouraging and is fueled by the growing demand for nutritious, allergy-friendly, and sustainable dietary options. Additionally, the increasing popularity of functional foods, gluten-free diets, and personalized nutrition offers opportunities for innovation, exemplified by the millet-based formulations developed in the present study.

## Data Availability

The raw data supporting the conclusions of this article will be made available by the authors, without undue reservation.
